# Neutrophils unveiled in chronic lymphocytic leukemia

**DOI:** 10.3389/fimmu.2025.1609754

**Published:** 2025-08-20

**Authors:** Sheighlah McManus, Priyanka Khare, Maria Teresa S. Bertilaccio

**Affiliations:** ^1^ Department of Leukemia, The University of Texas MD Anderson Cancer Center, Houston, TX, United States; ^2^ The University of Texas MD Anderson Cancer Center, UTHealth Houston Graduate School of Biomedical Sciences, Houston, TX, United States

**Keywords:** neutrophils, NETs, CLL, immune dysfunction, infection, disease progression

## Abstract

This review explores neutrophils’ roles in chronic lymphocytic leukemia (CLL), highlighting their functions within the immune system. While neutrophils are known for fighting infections, their altered behavior in CLL significantly impacts disease progression. This review notes the reduced phagocytic abilities of neutrophils and the increased formation of neutrophil extracellular traps (NETs) in patients with CLL. It also examines the effects of CLL treatments, including chemotherapy, immunotherapy and targeted therapies, on neutrophils’ count and function, stressing the need for improved strategies to manage therapy-induced immune dysfunction. This review also provides detailed information about the interactions between neutrophils and other immune elements in CLL microenvironment, providing insights for developing therapeutic approaches that can restore immune function and improve patients’ quality of life.

## 1 Introduction

**Figure 1:**
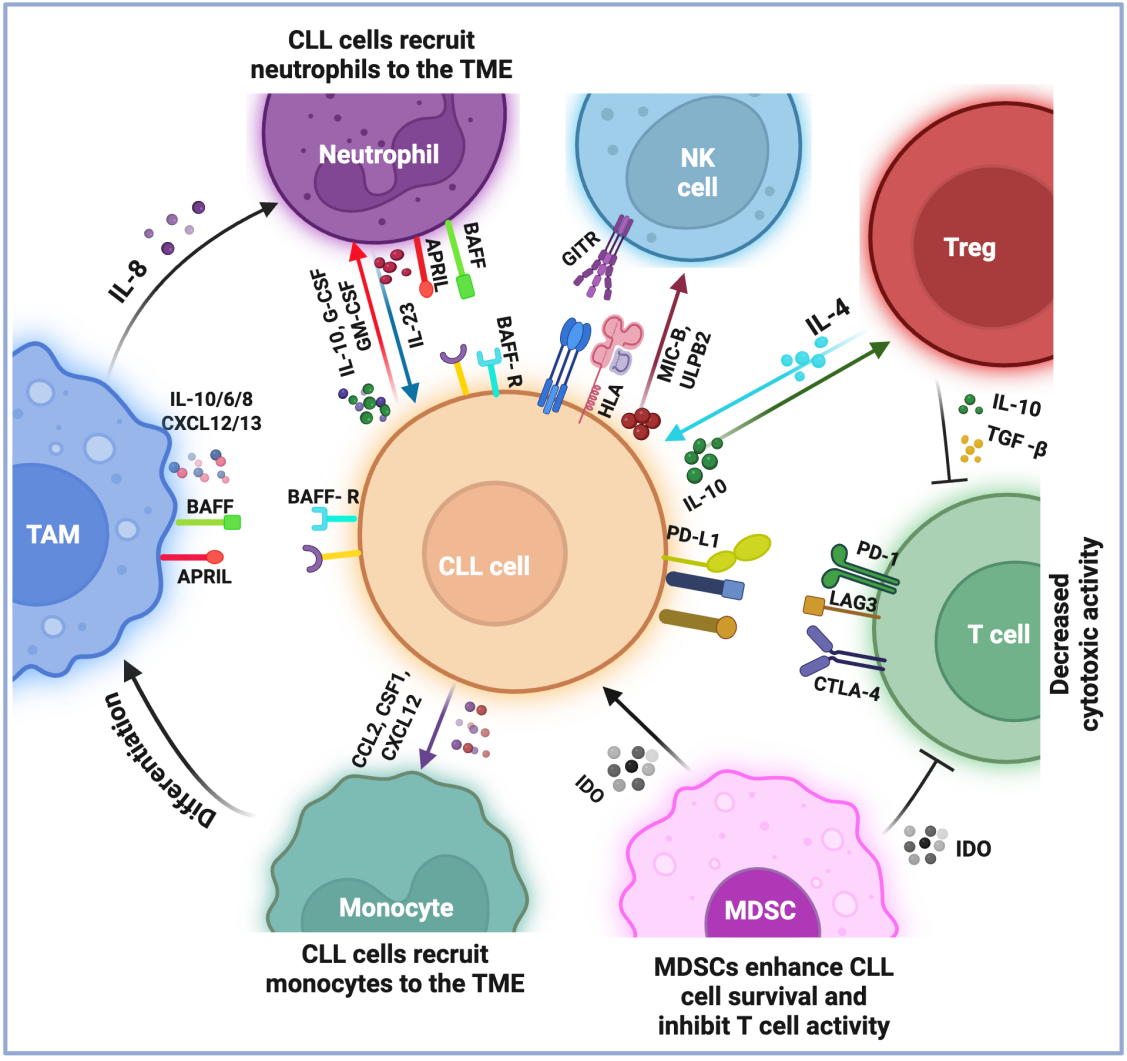
Current understandings of the immune microenvironment of chronic lymphocytic leukemia. The immune microenvironment of chronic lymphocytic leukemia (CLL) consists of lymphoid and myeloid cells, which play a role in the survival and growth of leukemia cells. T cells are generally exhausted and show decreased cytotoxic activity, marked by the expression of specific proteins such as PD1, CTLA-4, and LAG3. This exhaustion is further supported by immunosuppressive cells that inhibit T-cell activation by expressing particular molecules (i.e., IL-10 and TGFβ by Tregs and IDO by MDSCs). Monocytes are recruited to the microenvironment and transform into tumor-associated macrophages (TAMs), which produce molecules (i.e., BAFF, APRIL, IL-10/6/8, and CXCL12/13) that help CLL cells survive and grow. Neutrophils in the microenvironment also provide support for leukemia cell survival. CLL cells evade NK cell surveillance by expressing HLA class I molecules on their surface and by secreting MIC-B and ULPB2, which compromise NK function. Additionally, the TNFR receptor, GITR, axis with GITRL causes the release of TNF, IL6, and IL8, which act as survival factors for CLL cells. Overall, these interactions between different types of immune cells in the CLL microenvironment reduce the immune system’s ability to combat the leukemic cells, thus contributing to the persistence of the disease.

Chronic lymphocytic leukemia (CLL) is characterized by not only the accumulation of malignant B cells in the blood and lymphoid tissues but also a complex interplay of various immune cells within the tumor microenvironment [TME; **Figure 1** (1)]. The TME is an active and dynamic network that encompasses interactions between lymphoid and myeloid cells. Each of these cells plays a specific role in the progression of CLL, contributing to the immune evasion and dysfunction observed in this disease. The interactions among immune cells are mediated by various cytokines and chemokines that influence their behavior. This interplay shapes the immune response in CLL, aiding clonal expansion and establishing a supportive niche for leukemic cells (2). Among the immune cells of CLL TME, neutrophils play a critical role.

Neutrophils, the most abundant immune cells in the bloodstream, are a vital component of the innate immune system by serving as the body’s first line of defense against infections and inflammation (3). However, recent research has unveiled a more intricate and multifaceted role for neutrophils, particularly in the context of cancer (4). Emerging studies suggest that neutrophils can exhibit both pro-tumorigenic and anti-tumorigenic activities, complicating our understanding of their role in various disease states (5). They are also involved in various physiological and pathological conditions, including chronic inflammation, autoimmune diseases, diabetes, and thrombosis (6). Notably, neutrophils significantly influence the TME, affecting cancer progression, treatment response, and prognosis.

In patients with CLL, microbial infections are a leading cause of morbidity and mortality because of immune dysfunction caused and maintained by the disease itself (7–10). Additionally, treatments for CLL can alter immune responses and increase infection risk (9). As an example, the CLL14 (11) and MURANO (12) clinical studies reported adverse events such as neutropenia and various infections, including aspergillus pneumonia, herpes pharyngitis, and candida esophagitis (13). Understanding neutrophil biology is crucial because these cells are essential for patients’ defense against pathogens.

Overall, this evidence highlights how neutrophils contribute to disease progression and how this innate immune cell type impacts the intrinsic and therapy-induced immune dysfunction. Therefore, this literature review describes the intricate role of the immune microenvironment in CLL, with a focus on neutrophils and their functions. An enhanced understanding of these innate immune cells can be used to identify novel therapeutically targetable pathways that could restore proper immune function, thereby improving patients’ outcomes and quality of life.

## 2 Immune dysfunction in CLL

Previous studies identified two prevalent themes as the causes of the significant changes affecting the immune system of patients with CLL (14–16). First, intrinsic immune dysfunction arises directly from the disease itself (14). CLL disrupts the normal functioning of the adaptive immune response by altering the activity of essential lymphoid cells (i.e., B and T cells), which compromises the immune system’s ability to fight infections and respond effectively to pathogens. Second, the immune dysfunction in CLL patients can be worsened by various treatments, including chemotherapy (17, 18), immunotherapy (15), and targeted therapy (9, 16), which may inadvertently suppress the immune response or have other unintended consequences, such as increased susceptibility to infections. Consequently, patients with CLL often face a dual challenge of managing their disease while dealing with the repercussions of their treatment on their overall immune health.

### 2.1 Intrinsic immune dysfunction

Intrinsic immune dysfunction in CLL refers to the inherent alterations and impairments within the immune system that arise during the development and progression of the disease itself. These dysfunctions significantly contribute to the pathogenesis of CLL and its clinical manifestations, leading to a compromised immune response and an increased risk of infection and disease progression (19–23). For instance, leukemic B cells exhibit alterations that cause their accumulation in the blood and lymphoid tissues, which cause defects in their functional capabilities.

B cells naturally recognize specific antigens through the B-cell receptor (BCR), which consists of antigen-binding surface immunoglobulins and accessory molecules that interact with their corresponding antigens, leading to cell activation and antibody production. B-cell antibody production includes V(D)J recombination, somatic hypermutation, and class switching processes. In CLL, defects in antibody production and class switching often occur (24–26). The presence of dysfunctional B cells can reduce the ability to generate protective antibodies, leaving patients vulnerable to infections (27). BCR signaling is constitutively activated in CLL cells, whether this is attributable to self-antigens or environmental factors remains unclear (16–19). Nevertheless, signal transduction through the BCR activates pathways that promote the survival and growth of leukemic B cells, thereby facilitating disease progression.

Furthermore, patients with CLL have been shown to have an imbalance in the ratio of cluster of differentiation 4 (CD4)^+^ to CD8^+^ T cells (28). Although studies have documented a higher number of CD8^+^ T cells in CLL patients compared with age-matched healthy individuals, these cells often express exhaustion markers (i.e., programmed death cell protein 1[PD-1], cytotoxic T-lymphocyte associated protein 4 [CTLA-4], and lymphocyte-activation gene 3 [LAG-3]) and demonstrate reduced cytotoxic activity, which is perpetuated by improper immune synapse formation (29–31). Thus, CD8^+^ T cells often fail to release the cytotoxic granules necessary for eliminating leukemic cells. CLL patients often have a high frequency of CD4^+^ regulatory T cells (Treg). Treg cells are associated with hindered anti-tumor responses and immunosuppression, as they release immunosuppressive molecules (i.e., interleukin [IL]-10 and transforming growth factor beta [TGF-β]) that reduce CD8^+^ T-cell activation. Previous studies have demonstrated that removing Tregs leads to effective anti-tumor responses in animal models of CLL (32–34).

Natural killer (NK) cells, which are innate immune cells responsible for anti-tumor and anti-viral responses, are also present in the immune microenvironment of CLL. NK cell functions include degranulation, cytotoxicity, and the release of cytokines (35, 36). In CLL, NK cells’ cytolytic activity is impaired owing to defects in their cytotoxic machinery, and leukemic B cells employ immune evasion strategies to escape detection by NK cells (37).

Myeloid cells constitute a significant portion of the immune microenvironment in CLL. Nurse-like cells (NLCs) share lineage and functional similarities to tumor-associated macrophages (TAMs) and have been identified as CLL-specific TAMs (38). NLCs express stromal cell-derived factor 1 (SDF-1), which attaches to leukemic B cells and downregulates its receptor, C-X-C chemokine receptor type 4 (CXCR4), thereby protecting these cells from spontaneous apoptosis. NLCs also release B-cell activating factor (BAFF) and a proliferation-inducing ligand (APRIL), both of which contribute to the survival of CLL cells (39, 40). Another important myeloid cell component of the CLL TME are monocytes, which include CD14^++^ CD16^-^ classical, CD14^+^ CD16^++^ non-classical, and CD14^++^ CD16^+^ intermediate subsets (41, 42). The recruitment of monocytes to the TME depends on CLL cells’ expression of C-C-chemokine receptor type 2 (CCR2) and several monocyte-attracting chemokines such as C-C-motif ligand 2 (CCL2) and CXCL10 (43, 44). When adoptively transferred into humanized MISTRG mice, monocyte subsets from CLL patients differentiate into TAMs (45). Monocytic myeloid-derived suppressor cells (M-MDSCs) are so named because of their common myeloid origin and immunoregulatory properties (46, 47). In CLL patients, an increase in M-MDSCs in the peripheral blood (PB) is associated with a poor prognosis (48, 49). Additionally, in CLL patients, M-MDSCs suppress T cell function and stimulate the production of Tregs. Lastly, CLL cells induce the transition of monocytes into M-MDSCs, suggesting cellular communication between CLL cells, M-MDSCs, and Tregs (50).

The intrinsic immune dysfunction observed in CLL results from a complex interplay of factors that collectively compromise the immune system’s ability to respond effectively to the disease. By understanding these intrinsic mechanisms, researchers and clinicians can develop more effective strategies to restore immune function and enhance therapeutic approaches.

### 2.2 Therapy-induced immune dysfunction

Although chemotherapy, immunotherapy, and targeted therapy are vital for controlling CLL progression and improving outcomes, they often have significant side effects. A primary concern is these therapies’ ability to severely compromise the immune system and increase the risk of infection (9, 15, 17). Moreover, such therapy-induced immune dysfunction affects the immune system’s ability to perform essential surveillance, hindering its capability to detect and eliminate residual cancer cells, potentially causing relapse or disease progression.

#### 2.2.1 Chemotherapy

Commonly used chemotherapy drugs for CLL include alkylating agents, such as chlorambucil and cyclophosphamide, and purine analogs, such as fludarabine (18, 51–53). These drugs can lead to significant immune dysfunction. CLL patients receiving chlorambucil often experience bacterial infections, particularly in the respiratory system (54). Chlorambucil treatment can also cause lymphopenia, which significantly impacts the number of T cells, resulting in weakened adaptive immune responses (16). Patients receiving chlorambucil commonly have infections, including respiratory tract infections, bacterial pneumonia, and opportunistic infections (55). Cyclophosphamide combined with other agents, such as fludarabine, has been associated with neutropenia in CLL patients (56).

CLL patients treated with fludarabine alone have a heightened risk of infections, particularly if they previously received treatment for CLL, have advanced disease, or have a low neutrophil count (17). Of note, compared with chlorambucil, fludarabine is associated with a greater degree of neutropenia (52). Furthermore, purine analog-based treatment for CLL is associated with an increased risk of pneumonia, herpes simplex virus infection, and infections from other pathogens, such as *Pneumocystis jirovecii* (a fungal pathogen), *Listeria monocytogenes* (a bacterial pathogen), or cytomegalovirus (CMV; a viral pathogen) (17, 57).

#### 2.2.2 Immunotherapy

The immunotherapy strategies used to treat CLL were reviewed previously (58). The introduction of rituximab, a chimeric monoclonal antibody targeting CD20, combined with chemotherapy, improved patient survival and paved the way for immunotherapy in CLL (18, 53, 56, 58). However, rituximab reduces NK cells’ ability to mediate antibody-dependent cellular cytotoxicity against CLL cells (59). Newer generations of anti-CD20 monoclonal antibodies, such as obinutuzumab and ofatumumab, have significantly improved CD20-based immunotherapy (60, 61), but the spectrum of infections patients experience while on these medications is similar to that seen with rituximab. It has been reported that patients with comorbidities receiving obinutuzumab have higher rates of neutropenia than control arms (60). Untreated CLL patients show similar results when treated with ofatumumab as their initial therapy. Immune impairment remains a concern with anti-CD20 monotherapy, as cases of fatal reactivation of hepatitis B virus (HBV) and progressive multifocal leukoencephalopathy (PML) have been reported (62, 63). Additionally, late-onset neutropenia (LON) is a common side effect after the administration of anti-CD20 monoclonal antibodies (mAbs), occurring four or more weeks after the last dose (64–67). Patients with LON may develop subsequent infections, posing a challenge for clinicians and requiring close monitoring during and after therapy.

Another type of immunotherapy, CD19-targeted chimeric antigen receptor (CAR) T-cell therapy, is available for the treatment of CLL. While this therapy effectively eliminates CD19^+^ CLL cells, it also destroys normal B cells, resulting in B-cell aplasia (68). This condition can persist as long as CAR T cells continue to target CD19^+^ cells. The absence of functional B cells impairs the humoral immune response, thereby increasing susceptibility to infections.

#### 2.2.3 Targeted therapy

Targeted therapies, which selectively inhibit crucial signaling pathways responsible for survival and proliferation, have transformed the treatment landscape of CLL. Kinase inhibitors like phosphatidylinositol 3-kinase (PI3K) inhibitors (e.g., idelalisib, duvelisib) (69) and Bruton’s tyrosine kinase (BTK) inhibitors (e.g., ibrutinib, pirtobrutinib) (70) specifically target downstream BCR signaling pathways. B-cell lymphoma 2 (BCL-2) inhibitors, such as venetoclax, have also been integrated into CLL therapy (71). Despite their effectiveness, these interventions can exacerbate immune dysfunction (72, 73).

In a retrospective analysis of 378 patients with lymphoid cancers, Varughese et al. found that 53.3% of those receiving ibrutinib as monotherapy developed invasive bacterial infections, and 37.2% developed invasive fungal infections (10). This and other studies’ findings suggest that ibrutinib alters neutrophils’ ability to respond to infections. Blez et al. found that Ibrutinib reduces neutrophils’ reactive oxygen species (ROS) production, impairs their phagocytosis ability, and alters their cytokine release in response to Aspergillus fumigatus, a type of fungus (74). In a complementary study, Risnik et al. found similar results, as well as a slight impairment in neutrophil extracellular trap (NET) formation and a reduction in the activation of γδ T cells (75). In addition, several clinical trials have shown that patients receiving BTK inhibitors, like ibrutinib, have an elevated risk of infection with *Aspergillus*, *Cryptococcus*, and *Pneumocystis jirovecii*, as well as decreased NK cell, monocyte, and macrophage function (16). Lastly, in a clinical trial where patients were treated with the PI3K inhibitor idelalisib or placebo in combination with bendamustine plus rituximab, patients receiving idelalisib had a higher rate of infections, including opportunistic infections such as CMV or P. *jirovecii* (76).

## 3 Neutrophil biology in homeostasis and in cancer

**Figure 2:**
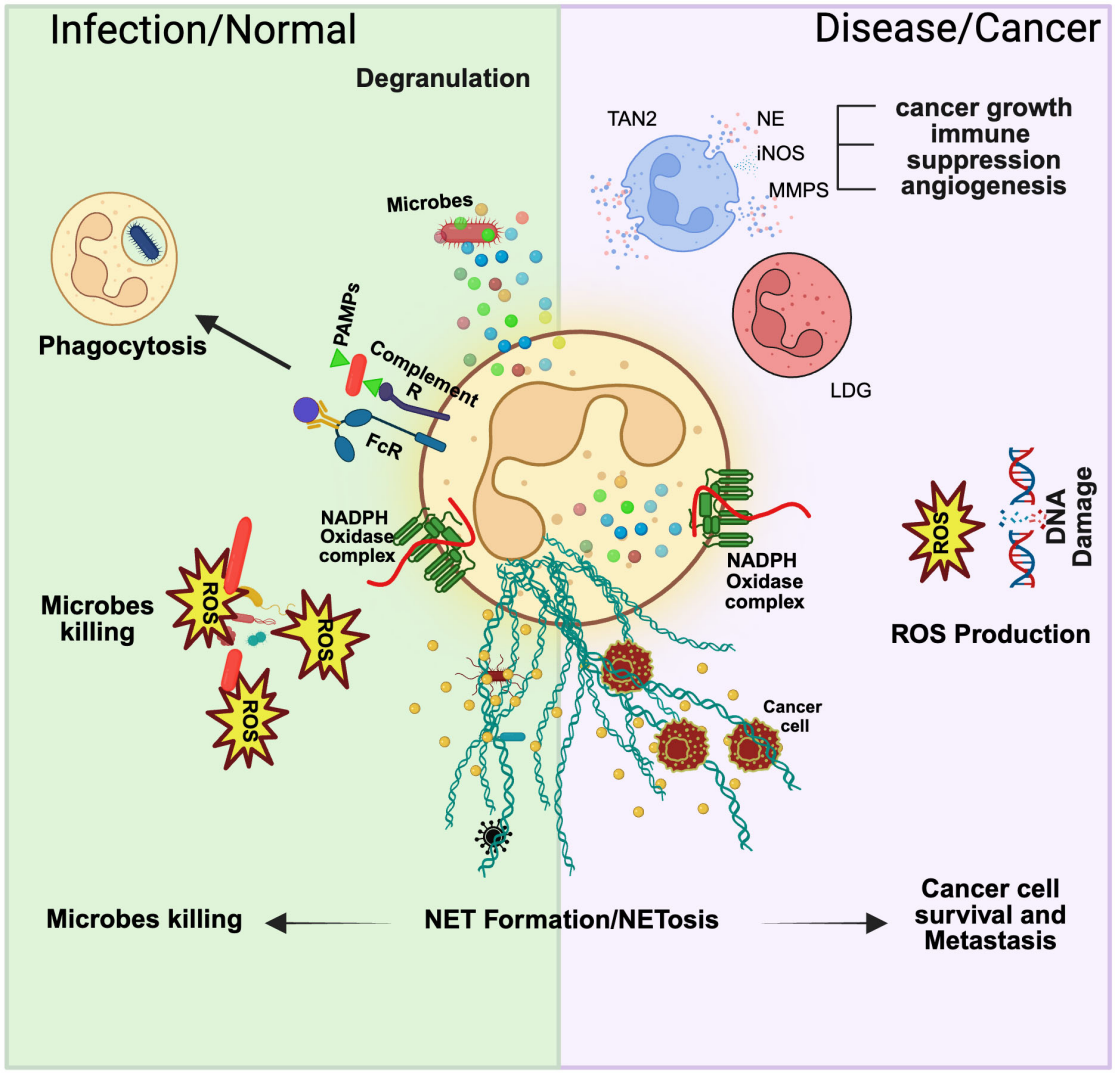
Neutrophil function in normal and disease states. Neutrophils display remarkable plasticity in their function, and the state of the host usually governs this. In normal physiological conditions, neutrophils can combat a pathogen via degranulation, phagocytosis, ROS production, and NETosis. In a disease or cancer context, these functions are modified, and various subsets of neutrophils can be identified in the bloodstream. Tumor-associated neutrophil type 2 (TAN2) is a cancer-specific neutrophil subset that can contribute to cancer growth, immune suppression, and angiogenesis. Low-density granulocytes (LDG) isolated from murine cancer models show overall reduced cytotoxic activity. Additionally, ROS production has been linked to DNA damage, which sparks carcinogenesis. Lastly, NET formation has been shown to favor cancer cell survival and metastasis by offering shielded protection during extravasation and migration.

Neutrophils are seen as primary responders to inflammation and infection. They rapidly initiate an immune response through degranulation, phagocytosis, ROS production, and NET formation (77–81). Once they eliminate a threat, they are quickly removed from the site, leading to a high turnover rate. However, emerging evidence suggests that neutrophils play roles beyond frontline defenders (**Figure 2**). The following section will examine their role in homeostasis and cancer.

### 3.1 Neutrophil development

Granulopoiesis, the process of neutrophil development, begins in the bone marrow where hematopoietic stem cells (HSC) differentiate into common myeloid progenitors [CMP (82)]. CMPs further differentiate into granulocyte-macrophage progenitors (GMP), which ultimately mature into neutrophils through a regulated process governed by various transcription factors. Recent research has identified unipotent cells within the neutrophil lineage; early neutrophil progenitors (eNePs) produce different types of neutrophils, categorized by function (83, 84). During the promyelocyte stage, neutrophils create primary granules such as neutrophil elastase (NE) and myeloperoxidase (MPO); during the myelocyte stage, they produce secondary granules. Once neutrophils develop into band cells, they form tertiary granules, such as arginase 1 (ARG1) and lysozyme. Fully differentiated neutrophils can leave the bone marrow in response to infection or inflammation (85–89). The bone marrow has a substantial reservoir of neutrophils. Granulocyte colony-stimulating factor (G-CSF) regulates granulopoiesis by inducing the proliferation of granulocytic precursors and influencing the release of mature neutrophils via the CXCR4-CXCL12 axis (88, 90).

Neutrophils are traditionally viewed as short-lived cells (88). However, recent research suggests that their lifespan in a disease condition is longer than previously believed (91). Specific transcription factors, such as GATA binding protein 1 (GATA1) and CCAAT/enhancer binding proteins (C/EBPs), regulate neutrophil production and their release into the bloodstream (92). The factors controlling circadian expression remain unknown. Signals from various cell types (i.e., bone marrow stromal cells, endothelial cells, and osteoblasts) collaborate to regulate the release or withholding of CXCL12 (92). Lastly, neutrophils can survive for extended periods following their activation by cytokines, inflammatory mediators, or microbial products (93). After completing their antimicrobial functions, neutrophils undergo apoptosis and are cleared by resident macrophages and dendritic cells (94).

### 3.2 Neutrophil function

Neutrophils rely on pre-existing effector molecules stored in intracellular granules to respond quickly to infection or inflammation (85). Degranulation is highly regulated and occurs hierarchically: secretory vesicles and tertiary granules are released first, followed by secondary and primary granules (95, 96). SNARE and Rab proteins regulate vesicle movement and degranulation (97).

Neutrophils produce ROS by activating nicotinamide adenine dinucleotide phosphate (NADPH) oxidase, which generates superoxide (O_2_-) and other ROS with potent antimicrobial activity (98). For instance, hydrogen peroxide (H_2_O_2_), in conjunction with MPO, produces hypochlorous acid (HOCl), a highly microbicidal substance (99). Some microbes can thwart ROS production by releasing toxins that prevent the assembly of the NADPH oxidase complex (100). Neutrophils have additional mechanisms to effectively combat pathogens.

Phagocytosis is facilitated by receptors that internalize particles into the phagosome, which is crucial for pathogen elimination (80). Neutrophils identify pathogens through pathogen-associated molecular patterns (PAMPs) or opsonins, which initiate signaling cascades that facilitate particle engulfment (101). The phagosome matures into a microbicidal vacuole known as the phagolysosome, which triggers ROS production by NADPH oxidase (102).

Neutrophils also form NETs to prevent the propagation of infection (81). During NETosis, a dynamic cell death program, neutrophils release chromatin fibers containing nuclear DNA along with antimicrobial proteins, which act as a framework to trap pathogens (103). However, unregulated NET formation can harm the host, contributing to noninfectious inflammatory diseases, such as cystic fibrosis and vasculitis. Excessive neutrophil recruitment or activation is linked to acute and chronic conditions such as myocardial infarction, stroke, systemic lupus erythematosus (SLE), and cancer (4), raising interest in neutrophils as a key player in various diseases.

### 3.3 Neutrophil phenotypes, plasticity, and function in cancer

Much of our understanding of neutrophils in cancer comes from studies of solid tumors in mouse models, and this information can act as a rationale for understanding the role neutrophils play in hematologic malignancies. Recent research has emphasized the crucial role of neutrophils in cancer, showing that the neutrophil-to-lymphocyte ratio (NLR) acts as a prognostic indicator in various solid tumors (104). For example, a high neutrophil count indicates a favorable prognosis in colon cancer patients, whereas lower neutrophil counts are associated with disease progression in breast cancer patients. This suggests that neutrophils can significantly influence the progression or regression of cancer (6, 105). NLR is emerging as a potentially useful prognostic indicator in hematologic malignancies, with studies suggesting that higher NLR values indicate increased inflammation and may be linked to poorer outcomes in patients (106). Neutrophils have been found to both combat and promote cancer by interacting with other immune cells within the TME and engaging in a range of functions, such as releasing substances that modulate the host response (107).

Neutrophils show remarkable adaptability in their role, either increasing or reducing inflammation. Their release of ROS is linked to DNA damage in lung cancer (108). In the TME, neutrophils are highly flexible, responding to signals that guide their activation and polarization, thereby influencing mechanisms that promote or hinder cancer development. It remains unclear whether neutrophils’ anti- or pro-tumor activities stem from specific activation under different microenvironmental conditions or if certain neutrophil subsets are predisposed to specific functions (3). Further research is needed to clarify the mechanisms behind the diverse functions of neutrophil subsets in both solid and hematologic cancers.

Patients with solid tumors and autoimmune diseases may have distinct subsets of mature neutrophils, including low-density granulocytes (LDGs (3)) and tumor-associated neutrophils (TANs) (5, 107). TANs are categorized as TAN1 and TAN2, which have opposing functions. TAN1 possesses anti-tumor properties; it releases cytokines such as IL-12 and tumor necrosis factor-alpha (TNF-α), which aid in the recruitment and activation of CD8^+^ T cells. In a murine model of lung carcinoma, TAN1 was associated with early tumor growth, whereas TAN2 emerged during tumor progression, influenced by the TME (109). In another study, the inhibition of TGF-β resulted in more cytotoxic and pro-inflammatory neutrophils, whose phenotype resembled that one of TAN1, whereas the presence of TGF-β leds to a pro-tumor phenotype. In human cancers, early-stage TANs acted as antigen-presenting cells (APCs), facilitating the priming, proliferation, and activation of anti-tumoral T cells (110).

LDGs, or low-density neutrophils (LDN), were first identified in autoimmune diseases as a pro-inflammatory subset linked to SLE progression. LDGs are thought to contribute to SLE pathogenesis by releasing NETs, which cause vascular damage and create autoantigens that induce autoimmunity (111). Increased LDGs are observed in SLE patients with a significant interferon (IFN) signature, a common marker of various autoimmune diseases, especially SLE (112, 113). In SLE, IFN- α production by plasmacytoid dendritic cells primes neutrophils to release NETs via Toll-like receptor 9 (TLR9) signaling (114, 115). NET levels positively correlate with lymphoma progression and childhood acute leukemia development (116, 117). In solid cancer, LDGs from tumor-bearing mice show reduced anti-tumor cytotoxicity, decreased phagocytosis, lower ROS production, increased anti-inflammatory activity, and tumor-promoting properties (118).

Further research clarifying the specific behaviors and roles of neutrophil subsets within the intricate and dynamic human TME would enhance our understanding of how these neutrophil subsets interact with the TME and how they might be therapeutically targeted.

## 4 Phenotype and functional abnormalities of neutrophils in CLL

Neutrophils play a crucial role in the immune response, during which their recruitment and phenotype undergo significant changes that impact their functionality. Unlike other immune cells, neutrophils are released from the bone marrow as fully functional entities, capable of recognizing and neutralizing pathogens (3). However, in cancer, these cells often enter the bloodstream prematurely (91). This premature release can lead to modifications in their function and phenotype, potentially compromising their effectiveness in immune defense.

Various cytokines and chemokines influence the recruitment of neutrophils to the CLL TME. For example, the pro-inflammatory factors IL-8 (a chemokine) and IL-17A (a cytokine), which are secreted by monocytes and tumor-associated macrophages, play a central role in attracting neutrophils to the TME (119–121). In CLL patients, elevated levels of these cytokines are correlated with poor prognostic outcomes, demonstrating that they not only facilitate neutrophil recruitment but also contribute to disease progression (122, 123).

The phenotype of neutrophils in CLL is characterized by significant alterations that reflect their activated state. Key surface markers associated with neutrophil function, including CD11b, CD64, and CD54, are upregulated on neutrophils isolated from patients with progressive CLL (124). This state of activation is often induced by the microenvironment, where cytokines such as IFN-γ are elevated (125). While this activated phenotype may enhance certain functions, it does not necessarily translate into improved pathogen neutralization, as neutrophils in CLL continue to exhibit functional defects. Podaza et al. investigated how CLL cells affect the neutrophil reprogramming. They identified a specific immunosuppressive subset of neutrophils in the circulation of CLL patients, defined as CD16^high^CD62L^dim^ neutrophils, whose formation depends on the secretion of IL-10 by leukemic B cells (126).

Recruited and activated neutrophils in CLL appear to have a reduced capacity for apoptosis. They show increased resistance to apoptosis *in vitro*, particularly when cultured with G-CSF (126). This survival advantage may lead to the accumulation of neutrophils in the microenvironment, potentially skewing their functional capabilities and contributing to the overall dysregulation of the immune response. Podaza et al. also assessed how G-CSF and GM-CSF extend the lifespan of neutrophils from healthy donors when exposed to CLL-conditioned media. They found that removing these growth factors neutralizes their antiapoptotic effects, showing that leukemic cell-produced factors are crucial for neutrophil prolonged survival. This evidence is clinically relevant, especially regarding the treatment of patient neutropenia. GM/G-CSF are commonly administered to boost neutrophil counts (127–130); however, a better understanding of the neutrophil-leukemic cell interactions may provide insights into the design of more effective treatment strategies with GM/G-CSF. Building upon this, G-CSF reduces neutrophil chemotaxis in stem cell transplant recipients for up to four weeks (131), underscoring the importance of exploring the roles of neutrophils in disease and treatment modalities.

Functionally, neutrophils isolated from CLL patients exhibit significant abnormalities that compromise their ability to effectively combat infections. For instance, neutrophils demonstrate impaired phagocytic killing of key non-opsonized bacterial pathogens, including *Staphylococcus aureus* and *Pseudomonas aeruginosa* (7). This impairment underscores the crucial role of neutrophils in the innate immune response, highlighting the challenges in managing bacterial infections in CLL patients. It has been shown that obinutuzumab can significantly modulate the phagocytic function of neutrophils in CLL by enhancing neutrophils’ expression of CD16b, which restores recognition and clearance of leukemic cells (132).

In addition to their phagocytic impairments, neutrophils in CLL exhibit reduced random migration and chemotaxis compared to healthy controls in response to stimuli such as *N*-formylmethionine-leucyl-phenylalanine (fMLP) and complement component 5a (C5a) (22). The impaired migration of neutrophils towards infection sites significantly hinders the immune response, making patients more vulnerable to infections.

Studies of neutrophils’ capacity to produce ROS in CLL have yielded various results. *In vitro* studies demonstrated that stimulation with fMLP or phorbol myristate acetate (PMA) can significantly enhance ROS generation by neutrophils from CLL patients compared with neutrophils from healthy controls (124). Conversely, *in vivo* studies showed no significant difference in ROS production by neutrophils from the bone marrow of Eµ-TCL1 mice compared with that by neutrophils from wild-type control mice (133). This difference emphasizes the need to develop more reliable research models that accurately mimic the complex immune microenvironment of CLL.

Finally, neutrophils from CLL patients release substantially more NETs following stimulation with PMA or a combination of TNF-α and lipopolysaccharide (LPS), compared to healthy donors (134). IL-8, whose levels are elevated in patients with CLL, has been shown to prime neutrophils to release NETs, thus suggesting a potential mechanism through which these cells may inadvertently support tumor progression while attempting to exert antimicrobial effects (121). These findings indicate that the leukemia microenvironment can influence dysfunctions specific to neutrophils, adding further complexity to the immune landscape in CLL.

## 5 Crosstalk between neutrophils and the immune microenvironment in CLL

Neutrophils play a role in regulating the adaptive immune response by directly interacting with T cells as antigen-presenting cells (APCs) and releasing various mediators that modulate the immune response (85). However, we have a limited understanding of how neutrophils in CLL interact with T cells directly. Podaza et al. documented that neutrophils isolated from patients with CLL more effectively inhibit T cell activation than those isolated from healthy donors following stimulation with phytohemagglutinin (PHA) (126). The direct mechanism of this inhibition remains unexplored. In addition, Gora et al. demonstrated that Treg cells contribute to the immunosuppressive phenotype of neutrophils *in vivo* through the modulation of essential markers, including CD62L and IL-4R (135). In this study, the depletion of Tregs caused the restoration of the functional neutrophil immunophenotypic profile. Other studies have indicated that neutrophils can express immunosuppressive markers, such as programmed death-ligand 1 (PD-L1), which inhibits T-cell activation, suggesting that neutrophils play an immunosuppressive role in their relationship with T cells (136). Additionally, NETs may induce apoptosis of both CD8^+^ and CD4^+^ T cells, leading to reduced immunity (137). Whether and, if so, at what disease stage this immunosuppressive activity is present in CLL remains to be investigated.

We have a better understanding of the interaction between neutrophils and leukemic B cells, although not all aspects have been completely elucidated. CD16^high^CD62L^dim^ immunosuppressive neutrophils have been shown to play a significant role in promoting B-cell maturation and survival by binding to BAFF and activating downstream pathways, including the nuclear factor kappa-light-chain-enhancer of activated B cells (NF-κβ) -dependent pathway, the non-canonical PI3K pathway, and protein B/mammalian target of rapamycin (AKT/mTOR) signaling pathway. At the opposite end of this interaction, leukemic B cells secrete G-CSF and granulocyte-macrophage colony-stimulating factor (GM-CSF), which increases the lifespan of neutrophils by upregulating the anti-apoptotic protein, Bcl-2-related protein A1 (BFL-1) (126). In a study with Eµ-TCL1 mice, neutrophils were predominantly found in the spleen’s red pulp and marginal zone (MZ). Splenic peri-MZ neutrophils (also known as B-cell helper neutrophils (138)) expressed BAFF, APRIL, IL-21, and chemokines such as CXCL12 and CXCL13, which induce the immunoglobulin class switching, somatic hypermutation, and activation of MZ B cells (133). Depleting neutrophils in these mice significantly reduced the splenic leukemic burden (133). Further research is necessary to fully comprehend the direct impact of neutrophils on the adaptive immune response.

Although neutrophils’ interactions with the myeloid compartment in CLL are largely unexplored, it is likely that they interact indirectly with monocytes, macrophages, and MDSCs. Monocytes in CLL have been shown to produce IL-8, which neutrophils utilize to generate NETs; IL-8 is also involved in neutrophils’ chemotaxis and activation, and can enhance their survival and cytokine production (121). Furthermore, MDSCs and TAMs/NLCs have been shown to produce IL-10 in CLL (139, 140). From studies in mouse models of lung disease, which have shown that IL-10 impedes neutrophil recruitment, thereby hindering the clearance of bacterial infections, we can infer that IL-10 likely negatively affects neutrophil behavior in CLL; however, the mechanisms by which it does so are unclear (141, 142). Much work is yet required to understand the full impact neutrophils have on the immune microenvironment of CLL.

## 6 Targeting NET formation and neutrophil polarization

The role of NETs in cancer development varies depending on the TME. NETs have been found to delay spontaneous apoptosis in leukemic B cells from CLL patients and enhance the expression of activation markers. Various stimuli can initiate the formation of NETs through interactions with surface markers, such as cytokine receptors, TLRs, and damage-associated molecular pattern (DAMP) receptors (143). These receptors activate a series of cellular processes leading to the release of the chromatin-protein mixture into the extracellular space. Each of these steps represents a potential therapeutic target for regulating the tumorigenic role of NETs in solid tumors and eventually in B-cell malignancies. Hence, it is crucial to explore therapeutic strategies that not only effectively target the disease but also restore proper immune function (**Table 1**).

**Table 1:** Targeting NET formation and neutrophil polarization.

Molecule Name	Description	References
PAD4 Inhibitors		
BMS-P5	First PAD4 inhibitor; reduces NETosis and decreases DNA and CitH3 levels	144, 145, 146
JBI-589	Oral bioavailability; reduces NETosis and citrullination	144, 145, 147
Potential Targeted Delivery Method		
Nano-particle-mediated Sivelestat	Reduces serum NE, and other pro-inflammatory cytokines; it also decreases NET formation	148, 149
Alternative Uses of FDA-approved Medications		
Prostaglandin E2 (PGE2)	In neutrophils, it can regulate NET release	153, 154, 155
Disulfiram	FDA approved for alcohol use disorder, in neutrophils it blocks GSDMD, which is critical for NET formation	157, 158
Targeting Neutrophil Polarization		
	Expose primary neutrophils to the TAN1 cocktail to repolarize tumor-promoting cells	159

### 6.1 PAD4 inhibitors

The protein arginine deiminase (PAD) family consists of a group of enzymes that are responsible for the post-translational modification (PTM) of protein arginine residues through deamination or demethylation, leading to the formation of citrulline. PAD4 is the only member of the family with a nuclear localization sequence. PAD4 competes with other nuclear enzymes to modify multiple arginine residues in histone H3 and H4. The citrullination of histone H3 (CitH3) has been linked to NET formation, highlighting the potential significance of targeting PAD4 in pathological conditions associated with excessive NET formation, such as cancer (144, 145).

BMS-P5 was the first PAD4 inhibitor to be developed. Li et al. demonstrated that administering BMS-P5 to human multiple myeloma (MM) cell lines reduced NETosis. Furthermore, this compound inhibited NET formation in primary bone marrow cells obtained from MM patients. Subsequently, *in vivo* studies revealed a significant reduction in DNA and CitH3 in a BMS-P5-treated mouse model of MM (146).

JBI-589 is another promising selective PAD4 inhibitor, notable for its oral bioavailability. Studies have shown that JBI-589 can stimulate immune responses against lung tumors, resulting in a substantial delay in tumor progression in lung cancer. Like BMS-P5, JBI-589 exhibited a PAD4-mediated preventative effect against NET formation and citrullination in murine neutrophils and arthritis models (147). PAD4 inhibitors could offer a novel approach to treat CLL and restore the proper function of neutrophils by disrupting pro-survival signals that leukemic B cells receive from NETs in the TME.

### 6.2 Nanoparticle-mediated sivelestat

Nanoparticles have emerged as a promising strategy for the targeted delivery of compounds to cells. Sivelestat was first used to target acute lung injury caused by systemic inflammatory response syndrome (SIRS (148)). This compound specifically inhibits the NE stored in neutrophil granules. Cruz et al. demonstrated the efficacy of nanoparticle-mediated sivelestat delivery in inhibiting NET formation (149). They noted a significant decrease in serum levels of NE and other proinflammatory cytokines; furthermore, treatment with nanoparticle-mediated sivelestat protected mice against endotoxic shock. Nanoparticle-mediated agents could serve as innovative therapeutic options able to reduce off-target effects, circumvent resistance mechanisms, and enhance drug effectiveness (150–152). These potential benefits could aid patients with CLL.

### 6.3 PGE2

Prostaglandin E2 (PGE2) is an endogenous protein with oxytocic properties that is widely seen in various clinical settings, including gastrointestinal cancer (153, 154). Studies with patients who have received autologous and allogeneic hematopoietic stem cell transplants have shown that PGE2 can inhibit NET formation through the induction of protein kinase A and Epac (155). In neutrophils, PGE2 effectively regulates NET release via autophagy induction, highlighting this compound’s ability to regulate NET formation and release. Besides its role in NET formation, PGE2 is linked to several pro-tumor mechanisms, including suppressing antitumor immunity, regulating tumor immune evasion, and promoting tumor progression (156). The specific role of PGE2 in CLL remains unclear; therefore, further research is needed to determine if inhibiting PGE2 could be an effective strategy.

### 6.4 Disulfiram

Disulfiram, a medication approved by the U.S. Food and Drug Administration for alcohol use disorder, inhibits aldehyde dehydrogenase. Studies have demonstrated its ability to block gasdermin D (GSDMD) in macrophages. In neutrophils, GSDMD is a pore-forming protein crucial for NET formation (157). Adrover et al. demonstrated that disulfiram effectively inhibited PMA-induced NET formation by neutrophils isolated from the peripheral blood of patients with COVID-19 (158); they also observed similar inhibitory effects in a mouse model of severe transfusion-related acute lung injury. In CLL, it is unexplored whether disulfiram can effectively reduce NET formation. A concern is its impact on macrophages, which may aggravate the immune dysfunction observed in patients. Overall, exploring alternative uses for FDA-approved drugs might present valuable opportunities for patients.

### 6.5 Neutrophil polarization-targeting therapy

Neutrophils exhibit remarkable plasticity, capable of assuming different phenotypes based on their maturation and surrounding environment (i.e., TAN1 or TAN2). Ohms et al. pioneered the attempt to polarize human neutrophils into distinct subsets. They exposed human neutrophils to either a TAN1 cocktail (containing LPS, IFN-β, and IFN-γ) or a TAN2 cocktail (containing L-lactate, adenosine, TGF-β, IL-10, PGE2, and G-CSF) and demonstrated that each respective cocktail could polarize mature neutrophils into tumor-associated subsets (159). Their findings suggest that interventions designed to polarize tumor-promoting neutrophils into anti-tumor subsets can be used to treat cancer and eventually CLL. Integrating these therapeutic strategies into the current treatment landscape for CLL might have the potential to significantly reduce neutrophil-mediated immune dysfunction in patients. By specifically targeting the formation of NETs and modulating the immune response, these approaches may not only hinder tumor progression but also improve overall patient survival rates.

## 7 Discussion

This review highlights the complex role of neutrophils in CLL, emphasizing the need to understand their multifaceted functions in both healthy and diseased states. Neutrophils, as the first responders of the innate immune system, play a crucial role in fighting infections and maintaining immune homeostasis. In CLL, however, their functions are significantly altered, which contributing to an immunocompromised state. This review identifies key abnormalities in neutrophil behavior, including decreased phagocytic abilities and increased formation of NETs, which not only fail to effectively eliminate pathogens but also may inadvertently support tumor progression.

This review also discussed how CLL affects neutrophils’ recruitment, phenotype, and function, thereby impacting their responsiveness to infection. Immunosuppressive neutrophil subsets, such as CD16^high^CD62L^dim^ neutrophils, might be involved in the deep immune dysfunction observed in CLL patients, highlighting the need for targeted therapeutic strategies aimed at immune restoration. The therapeutic landscape for CLL has evolved with the introduction of various treatment modalities, including chemotherapy, immunotherapy, and targeted therapies. While these treatments are essential for managing CLL, they often lead to immunosuppression, presenting a dual challenge for clinicians. Understanding the impact of these therapies on neutrophil function is critical, as it may inform patient management strategies to reduce the increased risk of infections. We emphasize the importance of exploring therapies that can enhance tumor-killing efficacy while addressing the underlying immune fitness. For instance, preclinical studies on the use of lenalidomide in combination with CD23-targeted CAR T-cell therapy showed how to enhance T-cell function and restore normal immune responses. Therapeutic combinations that address both intrinsic and therapy-induced immune dysfunctions should be further explored.

Advanced research is essential to elucidate the interactions between neutrophils and other immune cells in the CLL microenvironment. This knowledge could pave the way for innovative therapeutic approaches aimed at restoring proper immune function. The discovery of neutrophil-specific biomarkers that predict treatment responses and enable tailored therapies could significantly improve patient outcomes; targeting neutrophil-related immune dysfunction presents a promising avenue for advancing CLL treatment. Investigating the mechanisms that regulate neutrophils’ behavior and their contributions to the immune landscape may lead to interventions that enhance the effectiveness of existing therapies.

In conclusion, neutrophils play a critical yet often overlooked role in the immune dysfunction associated with CLL. Addressing their dysregulation and understanding their interactions within the immune microenvironment might lead to more effective treatment strategies and improved patient outcomes. As the field of immunotherapy continues to advance, integrating insights from neutrophil biology into therapeutic development will be essential for achieving sustained remission and enhancing the quality of life for CLL patients.

## References

[1] Kipps TJ Stevenson FK Wu CJ Croce CM Packham G Wierda WG. Chronic lymphocytic leukaemia. Nat Rev Dis Primers. (2017) 3:16096. doi: 10.1038/nrdp.2016.96, PMID: 28102226 PMC5336551

[2] Sun C Chen YC Martinez Zurita A Baptista MJ Pittaluga S Liu D. The immune microenvironment shapes transcriptional and genetic heterogeneity in chronic lymphocytic leukemia. Blood Adv. (2023) 7:145–58. doi: 10.1182/bloodadvances.2021006941, PMID: 35358998 PMC9811214

[3] Burn GL Foti A Marsman G Patel DF Zychlinsky A. The neutrophil. Immunity. (2021) 54:1377–91. doi: 10.1016/j.immuni.2021.06.006, PMID: 34260886

[4] Hedrick CC Malanchi I. Neutrophils in cancer: heterogeneous and multifaceted. Nat Rev Immunol. (2022) 22:173–87. doi: 10.1038/s41577-021-00571-6, PMID: 34230649

[5] Grecian R Whyte MKB Walmsley SR. The role of neutrophils in cancer. Br Med Bull. (2018) 128:5–14. doi: 10.1093/bmb/ldy029, PMID: 30137312 PMC6289220

[6] Amulic B Cazalet C Hayes GL Metzler KD Zychlinsky A. Neutrophil function: from mechanisms to disease. Annu Rev Immunol. (2012) 30:459–89. doi: 10.1146/annurev-immunol-020711-074942, PMID: 22224774

[7] Kontoyiannis DP Georgiadou SP Wierda WG Wright S Albert ND Ferrajoli A. Impaired bactericidal but not fungicidal activity of polymorphonuclear neutrophils in patients with chronic lymphocytic leukemia. Leuk Lymphoma. (2013) 54:1730–3. doi: 10.3109/10428194.2012.750723, PMID: 23163595 PMC3858983

[8] Lionakis MS Dunleavy K Roschewski M Widemann BC Butman JA Schmitz R. Inhibition of B cell receptor signaling by ibrutinib in primary CNS lymphoma. Cancer Cell. (2017) 31:833–43 e5. doi: 10.1016/j.ccell.2017.04.012, PMID: 28552327 PMC5571650

[9] Hilal T Gea-Banacloche JC Leis JF. Chronic lymphocytic leukemia and infection risk in the era of targeted therapies: Linking mechanisms with infections. Blood Rev. (2018) 32:387–99. doi: 10.1016/j.blre.2018.03.004, PMID: 29571669

[10] Varughese T Taur Y Cohen N Palomba ML Seo SK Hohl TM. Serious infections in patients receiving ibrutinib for treatment of lymphoid cancer. Clin Infect Dis. (2018) 67:687–92. doi: 10.1093/cid/ciy175, PMID: 29509845 PMC6093991

[11] Al-Sawaf O Robrecht S Zhang C Olivieri S Chang YM Fink AM. Venetoclax-obinutuzumab for previously untreated chronic lymphocytic leukemia: 6-year results of the randomized phase 3 CLL14 study. Blood. (2024) 144:1924–35. doi: 10.1182/blood.2024024631, PMID: 39082668 PMC11551846

[12] Al-Sawaf O. MURANO’s final conclusions: what we’ve learned, what’s next? Blood. (2025) 145:2674–6. doi: 10.1182/blood.2025028678, PMID: 40471626

[13] Seymour JF Kipps TJ Eichhorst BF D’Rozario J Owen CJ Assouline S. Enduring undetectable MRD and updated outcomes in relapsed/refractory CLL after fixed-duration venetoclax-rituximab. Blood. (2022) 140:839–50. doi: 10.1182/blood.2021015014, PMID: 35605176 PMC9412011

[14] Narkhede M Ujjani CS. Immune dysfunction and consequences in chronic lymphocytic leukemia. J Natl Compr Canc Netw. (2025) 23:1–7. doi: 10.6004/jnccn.2024.7090, PMID: 40073834

[15] Riches JC Ramsay AG Gribben JG. Immune dysfunction in chronic lymphocytic leukemia: the role for immunotherapy. Curr Pharm Des. (2012) 18:3389–98. doi: 10.2174/138161212801227023, PMID: 22591385

[16] Gargiulo E Teglgaard RS Faitova T Niemann CU. Immune dysfunction and infection - interaction between CLL and treatment: A reflection on current treatment paradigms and unmet needs. Acta Haematol. (2024) 147:84–98. doi: 10.1159/000533234, PMID: 37497921

[17] Anaissie EJ Kontoyiannis DP O’Brien S Kantarjian H Robertson L Lerner S. Infections in patients with chronic lymphocytic leukemia treated with fludarabine. Ann Intern Med. (1998) 129:559–66. doi: 10.7326/0003-4819-129-7-199810010-00010, PMID: 9758577

[18] Tam CS O’Brien S Wierda W Kantarjian H Wen S Do KA. Long-term results of the fludarabine, cyclophosphamide, and rituximab regimen as initial therapy of chronic lymphocytic leukemia. Blood. (2008) 112:975–80. doi: 10.1182/blood-2008-02-140582, PMID: 18411418 PMC3952498

[19] Hamblin AD Hamblin TJ. The immunodeficiency of chronic lymphocytic leukaemia. Br Med Bull. (2008) 87:49–62. doi: 10.1093/bmb/ldn034, PMID: 18755702

[20] Morrison VA. Infections in patients with leukemia and lymphoma. Cancer Treat Res. (2014) 161:319–49. doi: 10.1007/978-3-319-04220-6_11 24706230

[21] Morrison VA. Infectious complications of chronic lymphocytic leukaemia: pathogenesis, spectrum of infection, preventive approaches. Best Pract Res Clin Haematol. (2010) 23:145–53. doi: 10.1016/j.beha.2009.12.004, PMID: 20620978

[22] Itala M Vainio O Remes K. Functional abnormalities in granulocytes predict susceptibility to bacterial infections in chronic lymphocytic leukaemia. Eur J Haematol. (1996) 57:46–53. doi: 10.1111/j.1600-0609.1996.tb00489.x, PMID: 8698131

[23] Itala M Helenius H Nikoskelainen J Remes K. Infections and serum IgG levels in patients with chronic lymphocytic leukemia. Eur J Haematol. (1992) 48:266–70. doi: 10.1111/j.1600-0609.1992.tb01805.x, PMID: 1644158

[24] Hashimoto S Dono M Wakai M Allen SL Lichtman SM Schulman P. Somatic diversification and selection of immunoglobulin heavy and light chain variable region genes in IgG+ CD5+ chronic lymphocytic leukemia B cells. J Exp Med. (1995) 181:1507–17. doi: 10.1084/jem.181.4.1507, PMID: 7535340 PMC2191964

[25] Fais F Ghiotto F Hashimoto S Sellars B Valetto A Allen SL. Chronic lymphocytic leukemia B cells express restricted sets of mutated and unmutated antigen receptors. J Clin Invest. (1998) 102:1515–25. doi: 10.1172/JCI3009, PMID: 9788964 PMC509001

[26] Oscier DG Thompsett A Zhu D Stevenson FK. Differential rates of somatic hypermutation in V(H) genes among subsets of chronic lymphocytic leukemia defined by chromosomal abnormalities. Blood. (1997) 89:4153–60. doi: 10.1182/blood.V89.11.4153, PMID: 9166858

[27] Forconi F Moss P. Perturbation of the normal immune system in patients with CLL. Blood. (2015) 126:573–81. doi: 10.1182/blood-2015-03-567388, PMID: 26084672

[28] Gonzalez-Rodriguez AP Contesti J Huergo-Zapico L Lopez-Soto A Fernandez-Guizan A Acebes-Huerta A. Prognostic significance of CD8 and CD4 T cells in chronic lymphocytic leukemia. Leuk Lymphoma. (2010) 51:1829–36. doi: 10.3109/10428194.2010.503820, PMID: 20846097

[29] Scrivener S Goddard RV Kaminski ER Prentice AG. Abnormal T-cell function in B-cell chronic lymphocytic leukaemia. Leuk Lymphoma. (2003) 44:383–9. doi: 10.1080/1042819021000029993, PMID: 12688308

[30] Riches JC Davies JK McClanahan F Fatah R Iqbal S Agrawal S. T cells from CLL patients exhibit features of T-cell exhaustion but retain capacity for cytokine production. Blood. (2013) 121:1612–21. doi: 10.1182/blood-2012-09-457531, PMID: 23247726 PMC3587324

[31] Ramsay AG Johnson AJ Lee AM Gorgun G Le Dieu R Blum W. Chronic lymphocytic leukemia T cells show impaired immunological synapse formation that can be reversed with an immunomodulating drug. J Clin Invest. (2008) 118:2427–37. doi: 10.1172/JCI35017, PMID: 18551193 PMC2423865

[32] Beyer M Classen S Endl E Kochanek M Weihrauch MR Debey-Pascher S. Comparative approach to define increased regulatory T cells in different cancer subtypes by combined assessment of CD127 and FOXP3. Clin Dev Immunol. (2011) 2011:734036. doi: 10.1155/2011/734036, PMID: 21904560 PMC3166761

[33] Lad DP Varma S Varma N Sachdeva MU Bose P Malhotra P. Regulatory T-cells in B-cell chronic lymphocytic leukemia: their role in disease progression and autoimmune cytopenias. Leuk Lymphoma. (2013) 54:1012–9. doi: 10.3109/10428194.2012.728287, PMID: 23009220

[34] Lad D Hoeppli R Huang Q Garcia R Xu L Toze C. Regulatory T-cells drive immune dysfunction in CLL. Leuk Lymphoma. (2018) 59:486–9. doi: 10.1080/10428194.2017.1330475, PMID: 28573905

[35] Sivori S Vacca P Del Zotto G Munari E Mingari MC Moretta L. Human NK cells: surface receptors, inhibitory checkpoints, and translational applications. Cell Mol Immunol. (2019) 16:430–41. doi: 10.1038/s41423-019-0206-4, PMID: 30778167 PMC6474200

[36] Quatrini L Della Chiesa M Sivori S Mingari MC Pende D Moretta L. Human NK cells, their receptors and function. Eur J Immunol. (2021) 51:1566–79. doi: 10.1002/eji.202049028, PMID: 33899224 PMC9292411

[37] Sportoletti P De Falco F Del Papa B Baldoni S Guarente V Marra A. NK cells in chronic lymphocytic leukemia and their therapeutic implications. Int J Mol Sci. (2021) 22: 1566–1579. doi: 10.3390/ijms22136665, PMID: 34206399 PMC8268440

[38] Filip AA Cisel B Koczkodaj D Wasik-Szczepanek E Piersiak T Dmoszynska A. Circulating microenvironment of CLL: are nurse-like cells related to tumor-associated macrophages? Blood Cells Mol Dis. (2013) 50:263–70. doi: 10.1016/j.bcmd.2012.12.003, PMID: 23313631

[39] Tsukada N Burger JA Zvaifler NJ Kipps TJ. Distinctive features of “nurselike” cells that differentiate in the context of chronic lymphocytic leukemia. Blood. (2002) 99:1030–7. doi: 10.1182/blood.V99.3.1030, PMID: 11807009

[40] Nishio M Endo T Tsukada N Ohata J Kitada S Reed JC. Nurselike cells express BAFF and APRIL, which can promote survival of chronic lymphocytic leukemia cells via a paracrine pathway distinct from that of SDF-1alpha. Blood. (2005) 106:1012–20. doi: 10.1182/blood-2004-03-0889, PMID: 15860672 PMC1895149

[41] Bertilaccio MTS Zhang R Banerjee P Gandhi V. *In vitro* assay to study CLL and monocyte interactions. Methods Mol Biol. (2019) 1881:113–9. doi: 10.1007/978-1-4939-8876-1_9, PMID: 30350201

[42] Banerjee P Zhang R Ivan C Galletti G Clise-Dwyer K Barbaglio F. Trabectedin reveals a strategy of immunomodulation in chronic lymphocytic leukemia. Cancer Immunol Res. (2019) 7:2036–51. doi: 10.1158/2326-6066.CIR-19-0152, PMID: 31530560 PMC6891195

[43] van Attekum MH Eldering E Kater AP. Chronic lymphocytic leukemia cells are active participants in microenvironmental cross-talk. Haematologica. (2017) 102:1469–76. doi: 10.3324/haematol.2016.142679, PMID: 28775118 PMC5685246

[44] Gustafson MP Abraham RS Lin Y Wu W Gastineau DA Zent CS. Association of an increased frequency of CD14+ HLA-DR lo/neg monocytes with decreased time to progression in chronic lymphocytic leukaemia (CLL). Br J Haematol. (2012) 156:674–6. doi: 10.1111/j.1365-2141.2011.08902.x, PMID: 22050346 PMC3433277

[45] Zhang R Khare P Banerjee P Ivan C Schneider S Barbaglio F. The DLEU2/miR-15a/miR-16–1 cluster shapes the immune microenvironment of chronic lymphocytic leukemia. Blood Cancer J. (2024) 14:168. doi: 10.1038/s41408-024-01142-3, PMID: 39438453 PMC11496494

[46] Veglia F Sanseviero E Gabrilovich DI. Myeloid-derived suppressor cells in the era of increasing myeloid cell diversity. Nat Rev Immunol. (2021) 21:485–98. doi: 10.1038/s41577-020-00490-y, PMID: 33526920 PMC7849958

[47] Gabrilovich DI Bronte V Chen SH Colombo MP Ochoa A Ostrand-Rosenberg S. The terminology issue for myeloid-derived suppressor cells. Cancer Res. (2007) 67:425; author reply 6. doi: 10.1158/0008-5472.CAN-06-3037, PMID: 17210725 PMC1941787

[48] Gabrilovich DI Ostrand-Rosenberg S Bronte V. Coordinated regulation of myeloid cells by tumours. Nat Rev Immunol. (2012) 12:253–68. doi: 10.1038/nri3175, PMID: 22437938 PMC3587148

[49] Zarobkiewicz M Kowalska W Chocholska S Tomczak W Szymanska A Morawska I. High M-MDSC percentage as a negative prognostic factor in chronic lymphocytic leukaemia. Cancers (Basel). (2020) 12:2614. doi: 10.3390/cancers12092614, PMID: 32937740 PMC7563618

[50] Jitschin R Braun M Buttner M Dettmer-Wilde K Bricks J Berger J. CLL-cells induce IDOhi CD14+HLA-DRlo myeloid-derived suppressor cells that inhibit T-cell responses and promote TRegs. Blood. (2014) 124:750–60. doi: 10.1182/blood-2013-12-546416, PMID: 24850760

[51] Keating MJ McLaughlin P Plunkett W Robertson LE O’Brien S Gandhi V. Fludarabine–present status and future developments in chronic lymphocytic leukemia and lymphoma. Ann Oncol. (1994) 5 Suppl 2:79–83. doi: 10.1093/annonc/5.suppl_2.s79 8204523

[52] Rai KR Peterson BL Appelbaum FR Kolitz J Elias L Shepherd L. Fludarabine compared with chlorambucil as primary therapy for chronic lymphocytic leukemia. N Engl J Med. (2000) 343:1750–7. doi: 10.1056/NEJM200012143432402, PMID: 11114313

[53] Eichhorst B Fink AM Bahlo J Busch R Kovacs G Maurer C. First-line chemoimmunotherapy with bendamustine and rituximab versus fludarabine, cyclophosphamide, and rituximab in patients with advanced chronic lymphocytic leukaemia (CLL10): an international, open-label, randomised, phase 3, non-inferiority trial. Lancet Oncol. (2016) 17:928–42. doi: 10.1016/S1470-2045(16)30051-1, PMID: 27216274

[54] Jaksic B Brugiatelli M Krc I Losonczi H Holowiecki J Planinc-Peraica A. High dose chlorambucil versus Binet’s modified cyclophosphamide, doxorubicin, vincristine, and prednisone regimen in the treatment of patients with advanced B-cell chronic lymphocytic leukemia. Results of an international multicenter randomized trial. International Society for Chemo-Immunotherapy, Vienna. Cancer. (1997) 79:2107–14. doi: 10.1002/(SICI)1097-0142(19970601)79:11<2107::AID-CNCR7>3.0.CO;2-L, PMID: 9179056

[55] Robertson TI. Complications and causes of death in B cell chronic lymphocytic leukaemia: a long term study of 105 patients. Aust N Z J Med. (1990) 20:44–50. doi: 10.1111/j.1445-5994.1990.tb00370.x, PMID: 2322201

[56] Hallek M Fischer K Fingerle-Rowson G Fink AM Busch R Mayer J. Addition of rituximab to fludarabine and cyclophosphamide in patients with chronic lymphocytic leukaemia: a randomised, open-label, phase 3 trial. Lancet. (2010) 376:1164–74. doi: 10.1016/S0140-6736(10)61381-5, PMID: 20888994

[57] Anaissie E Kontoyiannis DP Kantarjian H Elting L Robertson LE Keating M. Listeriosis in patients with chronic lymphocytic leukemia who were treated with fludarabine and prednisone. Ann Intern Med. (1992) 117:466–9. doi: 10.7326/0003-4819-117-6-466, PMID: 1354425

[58] Freeman CL Gribben JG. Immunotherapy in chronic lymphocytic leukaemia (CLL). Curr Hematol Malig Rep. (2016) 11:29–36. doi: 10.1007/s11899-015-0295-9, PMID: 26857283 PMC4796351

[59] Werlenius O Aurelius J Hallner A Akhiani AA Simpanen M Martner A. Reactive oxygen species induced by therapeutic CD20 antibodies inhibit natural killer cell-mediated antibody-dependent cellular cytotoxicity against primary CLL cells. Oncotarget. (2016) 7:32046–53. doi: 10.18632/oncotarget.8769, PMID: 27097113 PMC5077995

[60] Goede V Fischer K Busch R Engelke A Eichhorst B Wendtner CM. Obinutuzumab plus chlorambucil in patients with CLL and coexisting conditions. N Engl J Med. (2014) 370:1101–10. doi: 10.1056/NEJMoa1313984, PMID: 24401022

[61] van Oers MH Kuliczkowski K Smolej L Petrini M Offner F Grosicki S. Ofatumumab maintenance versus observation in relapsed chronic lymphocytic leukaemia (PROLONG): an open-label, multicentre, randomised phase 3 study. Lancet Oncol. (2015) 16:1370–9. doi: 10.1016/S1470-2045(15)00143-6, PMID: 26377300

[62] Raisch DW Rafi JA Chen C Bennett CL. Detection of cases of progressive multifocal leukoencephalopathy associated with new biologicals and targeted cancer therapies from the FDA’s adverse event reporting system. Expert Opin Drug Saf. (2016) 15:1003–11. doi: 10.1080/14740338.2016.1198775, PMID: 27268272 PMC5020696

[63] Mitka M. FDA: Increased HBV reactivation risk with ofatumumab or rituximab. JAMA. (2013) 310:1664. doi: 10.1001/jama.2013.281115, PMID: 24150447

[64] Moore DC. Drug-induced neutropenia: A focus on rituximab-induced late-onset neutropenia. P T. (2016) 41:765–8., PMID: 27990078 PMC5132417

[65] Tesfa D Palmblad J. Late-onset neutropenia following rituximab therapy: incidence, clinical features and possible mechanisms. Expert Rev Hematol. (2011) 4:619–25. doi: 10.1586/ehm.11.62, PMID: 22077526

[66] Shimony S Bar-Sever E Berger T Itchaki G Gurion R Yeshurun M. Late onset neutropenia after rituximab and obinutuzumab treatment - characteristics of a class-effect toxicity. Leuk Lymphoma. (2021) 62:2921–7. doi: 10.1080/10428194.2021.1948037, PMID: 34284690

[67] Athni TS Barmettler S. Hypogammaglobulinemia, late-onset neutropenia, and infections following rituximab. Ann Allergy Asthma Immunol. (2023) 130:699–712. doi: 10.1016/j.anai.2023.01.018, PMID: 36706910 PMC10247428

[68] Todorovic Z Todorovic D Markovic V Ladjevac N Zdravkovic N Djurdjevic P. CAR T cell therapy for chronic lymphocytic leukemia: successes and shortcomings. Curr Oncol. (2022) 29:3647–57. doi: 10.3390/curroncol29050293, PMID: 35621683 PMC9139644

[69] Lampson BL Brown JR. The evolving use of phosphatidylinositol 3-kinase inhibitors for the treatment of chronic lymphocytic leukemia. Hematol Oncol Clin North Am. (2021) 35:807–26. doi: 10.1016/j.hoc.2021.03.009, PMID: 34174987 PMC8239250

[70] Tam C Thompson PA. BTK inhibitors in CLL: second-generation drugs and beyond. Blood Adv. (2024) 8:2300–9. doi: 10.1182/bloodadvances.2023012221, PMID: 38478390 PMC11117011

[71] Roberts AW Huang D. Targeting BCL2 with BH3 mimetics: basic science and clinical application of venetoclax in chronic lymphocytic leukemia and related B cell Malignancies. Clin Pharmacol Ther. (2017) 101:89–98. doi: 10.1002/cpt.553, PMID: 27806433 PMC5657403

[72] Nasillo V Lagreca I Vallerini D Barozzi P Riva G Maccaferri M. BTK inhibitors impair platelet-mediated antifungal activity. Cells. (2022) 11:1003. doi: 10.3390/cells11061003, PMID: 35326454 PMC8947638

[73] Korycka-Wolowiec A Wolowiec D Lawnicka H Robak T. Assessing adverse event burden in chronic lymphocytic leukemia treatment regimens: what’s best for patient quality of life? Expert Opin Drug Saf. (2025) 24:643–55. doi: 10.1080/14740338.2025.2471508, PMID: 39991898

[74] Blez D Blaize M Soussain C Boissonnas A Meghraoui-Kheddar A Menezes N. Ibrutinib induces multiple functional defects in the neutrophil response against Aspergillus fumigatus. Haematologica. (2019) 104:1203–13. doi: 10.3324/haematol.2019.219220, PMID: 31171644 PMC7012467

[75] Risnik D Elias EE Keitelman I Colado A Podaza E Cordini G. The effect of ibrutinib on neutrophil and gammadelta T cell functions. Leuk Lymphoma. (2020) 61:2409–18. doi: 10.1080/10428194.2020.1753043, PMID: 32306816

[76] Zelenetz AD Barrientos JC Brown JR Coiffier B Delgado J Egyed M. Idelalisib or placebo in combination with bendamustine and rituximab in patients with relapsed or refractory chronic lymphocytic leukaemia: interim results from a phase 3, randomised, double-blind, placebo-controlled trial. Lancet Oncol. (2017) 18:297–311. doi: 10.1016/S1470-2045(16)30671-4 28139405 PMC5589180

[77] Mayadas TN Cullere X Lowell CA. The multifaceted functions of neutrophils. Annu Rev Pathol. (2014) 9:181–218. doi: 10.1146/annurev-pathol-020712-164023, PMID: 24050624 PMC4277181

[78] Soehnlein O. Direct and alternative antimicrobial mechanisms of neutrophil-derived granule proteins. J Mol Med (Berl). (2009) 87:1157–64. doi: 10.1007/s00109-009-0508-6, PMID: 19641860

[79] Zeng MY Miralda I Armstrong CL Uriarte SM Bagaitkar J. The roles of NADPH oxidase in modulating neutrophil effector responses. Mol Oral Microbiol. (2019) 34:27–38. doi: 10.1111/omi.12252, PMID: 30632295 PMC6935359

[80] Rosales C Uribe-Querol E. Phagocytosis: A fundamental process in immunity. BioMed Res Int. (2017) 2017:9042851. doi: 10.1155/2017/9042851, PMID: 28691037 PMC5485277

[81] Yipp BG Petri B Salina D Jenne CN Scott BN Zbytnuik LD. Infection-induced NETosis is a dynamic process involving neutrophil multitasking *in vivo* . Nat Med. (2012) 18:1386–93. doi: 10.1038/nm.2847, PMID: 22922410 PMC4529131

[82] Ward AC Loeb DM Soede-Bobok AA Touw IP Friedman AD. Regulation of granulopoiesis by transcription factors and cytokine signals. Leukemia. (2000) 14:973–90. doi: 10.1038/sj.leu.2401808, PMID: 10865962

[83] Dinh HQ Eggert T Meyer MA Zhu YP Olingy CE Llewellyn R. Coexpression of CD71 and CD117 identifies an early unipotent neutrophil progenitor population in human bone marrow. Immunity. (2020) 53:319–34 e6. doi: 10.1016/j.immuni.2020.07.017, PMID: 32814027 PMC7942809

[84] Kwok I Becht E Xia Y Ng M Teh YC Tan L. Combinatorial single-cell analyses of granulocyte-monocyte progenitor heterogeneity reveals an early uni-potent neutrophil progenitor. Immunity. (2020) 53:303–18 e5. doi: 10.1016/j.immuni.2020.06.005, PMID: 32579887

[85] Rosales C. Neutrophils at the crossroads of innate and adaptive immunity. J Leukoc Biol. (2020) 108:377–96. doi: 10.1002/JLB.4MIR0220-574RR, PMID: 32202340

[86] Cowland JB Borregaard N. Granulopoiesis and granules of human neutrophils. Immunol Rev. (2016) 273:11–28. doi: 10.1111/imr.12440, PMID: 27558325

[87] Hager M Cowland JB Borregaard N. Neutrophil granules in health and disease. J Intern Med. (2010) 268:25–34. doi: 10.1111/j.1365-2796.2010.02237.x, PMID: 20497300

[88] Summers C Rankin SM Condliffe AM Singh N Peters AM Chilvers ER. Neutrophil kinetics in health and disease. Trends Immunol. (2010) 31:318–24. doi: 10.1016/j.it.2010.05.006, PMID: 20620114 PMC2930213

[89] Yvan-Charvet L Ng LG. Granulopoiesis and neutrophil homeostasis: A metabolic, daily balancing act. Trends Immunol. (2019) 40:598–612. doi: 10.1016/j.it.2019.05.004, PMID: 31256783

[90] Strydom N Rankin SM. Regulation of circulating neutrophil numbers under homeostasis and in disease. J Innate Immun. (2013) 5:304–14. doi: 10.1159/000350282, PMID: 23571274 PMC6741587

[91] Adrover JM McDowell SAC He XY Quail DF Egeblad M. NETworking with cancer: The bidirectional interplay between cancer and neutrophil extracellular traps. Cancer Cell. (2023) 41:505–26. doi: 10.1016/j.ccell.2023.02.001, PMID: 36827980 PMC10280682

[92] Aroca-Crevillen A Adrover JM Hidalgo A. Circadian features of neutrophil biology. Front Immunol. (2020) 11:576. doi: 10.3389/fimmu.2020.00576, PMID: 32346378 PMC7169427

[93] Lahoz-Beneytez J Elemans M Zhang Y Ahmed R Salam A Block M. Human neutrophil kinetics: modeling of stable isotope labeling data supports short blood neutrophil half-lives. Blood. (2016) 127:3431–8. doi: 10.1182/blood-2016-03-700336, PMID: 27136946 PMC4929930

[94] Greenlee-Wacker MC. Clearance of apoptotic neutrophils and resolution of inflammation. Immunol Rev. (2016) 273:357–70. doi: 10.1111/imr.12453, PMID: 27558346 PMC5000862

[95] Lacy P. Mechanisms of degranulation in neutrophils. Allergy Asthma Clin Immunol. (2006) 2:98–108. doi: 10.1186/1710-1492-2-3-98, PMID: 20525154 PMC2876182

[96] Lacy P Eitzen G. Control of granule exocytosis in neutrophils. Front Biosci. (2008) 13:5559–70. doi: 10.2741/3099, PMID: 18508605

[97] Ramadass M Catz SD. Molecular mechanisms regulating secretory organelles and endosomes in neutrophils and their implications for inflammation. Immunol Rev. (2016) 273:249–65. doi: 10.1111/imr.12452, PMID: 27558339 PMC6363536

[98] Dupre-Crochet S Erard M Nubetae O. ROS production in phagocytes: why, when, and where? J Leukoc Biol. (2013) 94:657–70. doi: 10.1189/jlb.1012544, PMID: 23610146

[99] Lin W Chen H Chen X Guo C. The roles of neutrophil-derived myeloperoxidase (MPO) in diseases: the new progress. Antioxidants (Basel). (2024) 13:132. doi: 10.3390/antiox13010132, PMID: 38275657 PMC10812636

[100] Nguyen GT Green ER Mecsas J. Neutrophils to the ROScue: mechanisms of NADPH oxidase activation and bacterial resistance. Front Cell Infect Microbiol. (2017) 7:373. doi: 10.3389/fcimb.2017.00373, PMID: 28890882 PMC5574878

[101] Ostrowski PP Grinstein S Freeman SA. Diffusion barriers, mechanical forces, and the biophysics of phagocytosis. Dev Cell. (2016) 38:135–46. doi: 10.1016/j.devcel.2016.06.023, PMID: 27459066

[102] Green JN Chapman ALP Bishop CJ Winterbourn CC Kettle AJ. Neutrophil granule proteins generate bactericidal ammonia chloramine on reaction with hydrogen peroxide. Free Radic Biol Med. (2017) 113:363–71. doi: 10.1016/j.freeradbiomed.2017.10.343, PMID: 29055823

[103] Fuchs TA Abed U Goosmann C Hurwitz R Schulze I Wahn V. Novel cell death program leads to neutrophil extracellular traps. J Cell Biol. (2007) 176:231–41. doi: 10.1083/jcb.200606027, PMID: 17210947 PMC2063942

[104] Heshmat-Ghahdarijani K Sarmadi V Heidari A Falahati Marvasti A Neshat S Raeisi S. The neutrophil-to-lymphocyte ratio as a new prognostic factor in cancers: a narrative review. Front Oncol. (2023) 13:1228076. doi: 10.3389/fonc.2023.1228076, PMID: 37860198 PMC10583548

[105] Liew PX Kubes P. The neutrophil’s role during health and disease. Physiol Rev. (2019) 99:1223–48. doi: 10.1152/physrev.00012.2018, PMID: 30758246

[106] Stefaniuk P Szymczyk A Podhorecka M. The neutrophil to lymphocyte and lymphocyte to monocyte ratios as new prognostic factors in hematological Malignancies - A narrative review. Cancer Manag Res. (2020) 12:2961–77. doi: 10.2147/CMAR.S245928, PMID: 32425606 PMC7196794

[107] Quail DF Amulic B Aziz M Barnes BJ Eruslanov E Fridlender ZG. Neutrophil phenotypes and functions in cancer: A consensus statement. J Exp Med. (2022) 219:e20220011. doi: 10.1084/jem.20220011, PMID: 35522219 PMC9086501

[108] Wculek SK Bridgeman VL Peakman F Malanchi I. Early neutrophil responses to chemical carcinogenesis shape long-term lung cancer susceptibility. iScience. (2020) 23:101277. doi: 10.1016/j.isci.2020.101277, PMID: 32619702 PMC7334367

[109] Mishalian I Bayuh R Levy L Zolotarov L Michaeli J Fridlender ZG. Tumor-associated neutrophils (TAN) develop pro-tumorigenic properties during tumor progression. Cancer Immunol Immunother. (2013) 62:1745–56. doi: 10.1007/s00262-013-1476-9, PMID: 24092389 PMC11028422

[110] Eruslanov EB. Phenotype and function of tumor-associated neutrophils and their subsets in early-stage human lung cancer. Cancer Immunol Immunother. (2017) 66:997–1006. doi: 10.1007/s00262-017-1976-0, PMID: 28283697 PMC5522629

[111] Denny MF Yalavarthi S Zhao W Thacker SG Anderson M Sandy AR. A distinct subset of proinflammatory neutrophils isolated from patients with systemic lupus erythematosus induces vascular damage and synthesizes type I IFNs. J Immunol. (2010) 184:3284–97. doi: 10.4049/jimmunol.0902199, PMID: 20164424 PMC2929645

[112] Rahman S Sagar D Hanna RN Lightfoot YL Mistry P Smith CK. Low-density granulocytes activate T cells and demonstrate a non-suppressive role in systemic lupus erythematosus. Ann Rheum Dis. (2019) 78:957–66. doi: 10.1136/annrheumdis-2018-214620, PMID: 31040119 PMC6585283

[113] Carmona-Rivera C Kaplan MJ. Low-density granulocytes: a distinct class of neutrophils in systemic autoimmunity. Semin Immunopathol. (2013) 35:455–63. doi: 10.1007/s00281-013-0375-7, PMID: 23553215 PMC4007274

[114] Garcia-Romo GS Caielli S Vega B Connolly J Allantaz F Xu Z. Netting neutrophils are major inducers of type I IFN production in pediatric systemic lupus erythematosus. Sci Transl Med. (2011) 3:73ra20. doi: 10.1126/scitranslmed.3001201, PMID: 21389264 PMC3143837

[115] Lande R Ganguly D Facchinetti V Frasca L Conrad C Gregorio J. Neutrophils activate plasmacytoid dendritic cells by releasing self-DNA-peptide complexes in systemic lupus erythematosus. Sci Transl Med. (2011) 3:73ra19. doi: 10.1126/scitranslmed.3001180, PMID: 21389263 PMC3399524

[116] Nie M Yang L Bi X Wang Y Sun P Yang H. Neutrophil extracellular traps induced by IL8 promote diffuse large B-cell lymphoma progression via the TLR9 signaling. Clin Cancer Res. (2019) 25:1867–79. doi: 10.1158/1078-0432.CCR-18-1226, PMID: 30446590

[117] Ostafin M Ciepiela O Pruchniak M Wachowska M Ulinska E Mrowka P. Dynamic changes in the ability to release neutrophil extraCellular traps in the course of childhood acute leukemias. Int J Mol Sci. (2021) 22:821. doi: 10.3390/ijms22020821, PMID: 33467555 PMC7829911

[118] Sagiv JY Michaeli J Assi S Mishalian I Kisos H Levy L. Phenotypic diversity and plasticity in circulating neutrophil subpopulations in cancer. Cell Rep. (2015) 10:562–73. doi: 10.1016/j.celrep.2014.12.039, PMID: 25620698

[119] Ferretti S Bonneau O Dubois GR Jones CE Trifilieff A. IL-17, produced by lymphocytes and neutrophils, is necessary for lipopolysaccharide-induced airway neutrophilia: IL-15 as a possible trigger. J Immunol. (2003) 170:2106–12. doi: 10.4049/jimmunol.170.4.2106, PMID: 12574382

[120] Flannigan KL Ngo VL Geem D Harusato A Hirota SA Parkos CA. IL-17A-mediated neutrophil recruitment limits expansion of segmented filamentous bacteria. Mucosal Immunol. (2017) 10:673–84. doi: 10.1038/mi.2016.80, PMID: 27624780 PMC5350071

[121] Risnik D Podaza E Almejun MB Colado A Elias EE Bezares RF. Revisiting the role of interleukin-8 in chronic lymphocytic leukemia. Sci Rep. (2017) 7:15714. doi: 10.1038/s41598-017-15953-x, PMID: 29146966 PMC5691131

[122] Kouzegaran S Siroosbakht S Farsad BF Rezakhaniha B Dormanesh B Behnod V. Elevated IL-17A and IL-22 regulate expression of inducible CD38 and Zap-70 in chronic lymphocytic leukemia. Cytometry B Clin Cytom. (2018) 94:143–7. doi: 10.1002/cyto.b.21487, PMID: 27718514

[123] Francia di Celle P Mariani S Riera L Stacchini A Reato G Foa R. Interleukin-8 induces the accumulation of B-cell chronic lymphocytic leukemia cells by prolonging survival in an autocrine fashion. Blood. (1996) 87:4382–9. doi: 10.1182/blood.V87.10.4382.bloodjournal87104382, PMID: 8639799

[124] Manukyan G Papajik T Gajdos P Mikulkova Z Urbanova R Gabcova G. Neutrophils in chronic lymphocytic leukemia are permanently activated and have functional defects. Oncotarget. (2017) 8:84889–901. doi: 10.18632/oncotarget.20031, PMID: 29156691 PMC5689581

[125] Buschle M Campana D Carding SR Richard C Hoffbrand AV Brenner MK. Interferon gamma inhibits apoptotic cell death in B cell chronic lymphocytic leukemia. J Exp Med. (1993) 177:213–8. doi: 10.1084/jem.177.1.213, PMID: 7678114 PMC2190861

[126] Podaza E Risnik D Colado A Elias E Almejun MB Fernandez Grecco H. Chronic lymphocytic leukemia cells increase neutrophils survival and promote their differentiation into CD16(high) CD62L(dim) immunosuppressive subset. Int J Cancer. (2019) 144:1128–34. doi: 10.1002/ijc.31762, PMID: 30178523

[127] Itala M Pelliniemi TT Remes K Vanhatalo S Vainio O. Long-term treatment with GM-CSF in patients with chronic lymphocytic leukemia and recurrent neutropenic infections. Leuk Lymphoma. (1998) 32:165–74. doi: 10.3109/10428199809059257, PMID: 10037012

[128] Mehta HM Malandra M Corey SJ. G-CSF and GM-CSF in neutropenia. J Immunol. (2015) 195:1341–9. doi: 10.4049/jimmunol.1500861, PMID: 26254266 PMC4741374

[129] Dale DC. Colony-stimulating factors for the management of neutropenia in cancer patients. Drugs. (2002) 62 Suppl 1:1–15. doi: 10.2165/00003495-200262001-00001, PMID: 12479591

[130] Spiekermann K Roesler J Emmendoerffer A Elsner J Welte K. Functional features of neutrophils induced by G-CSF and GM-CSF treatment: differential effects and clinical implications. Leukemia. (1997) 11:466–78. doi: 10.1038/sj.leu.2400607, PMID: 9096685

[131] Thunstrom Salzer A Niemiec MJ Hosseinzadeh A Stylianou M Astrom F Rohm M. Assessment of neutrophil chemotaxis upon G-CSF treatment of healthy stem cell donors and in allogeneic transplant recipients. Front Immunol. (2018) 9:1968. doi: 10.3389/fimmu.2018.01968, PMID: 30254629 PMC6141688

[132] Golay J Da Roit F Bologna L Ferrara C Leusen JH Rambaldi A. Glycoengineered CD20 antibody obinutuzumab activates neutrophils and mediates phagocytosis through CD16B more efficiently than rituximab. Blood. (2013) 122:3482–91. doi: 10.1182/blood-2013-05-504043, PMID: 24106207

[133] Gatjen M Brand F Grau M Gerlach K Kettritz R Westermann J. Splenic marginal zone granulocytes acquire an accentuated neutrophil B-cell helper phenotype in chronic lymphocytic leukemia. Cancer Res. (2016) 76:5253–65. doi: 10.1158/0008-5472.CAN-15-3486, PMID: 27488528

[134] Podaza E Sabbione F Risnik D Borge M Almejun MB Colado A. Neutrophils from chronic lymphocytic leukemia patients exhibit an increased capacity to release extracellular traps (NETs). Cancer Immunol Immunother. (2017) 66:77–89. doi: 10.1007/s00262-016-1921-7, PMID: 27796477 PMC11029506

[135] Goral A Sledz M Manda-Handzlik A Cieloch A Wojciechowska A Lachota M. Regulatory T cells contribute to the immunosuppressive phenotype of neutrophils in a mouse model of chronic lymphocytic leukemia. Exp Hematol Oncol. (2023) 12:89. doi: 10.1186/s40164-023-00452-9, PMID: 37817276 PMC10563345

[136] Langereis JD Pickkers P de Kleijn S Gerretsen J de Jonge MI Kox M. Spleen-derived IFN-gamma induces generation of PD-L1(+)-suppressive neutrophils during endotoxemia. J Leukoc Biol. (2017) 102:1401–9. doi: 10.1189/jlb.3A0217-051RR, PMID: 28974543

[137] Kaltenmeier C Yazdani HO Morder K Geller DA Simmons RL Tohme S. Neutrophil extracellular traps promote T cell exhaustion in the tumor microenvironment. Front Immunol. (2021) 12:785222. doi: 10.3389/fimmu.2021.785222, PMID: 34899751 PMC8652262

[138] Puga I Cols M Barra CM He B Cassis L Gentile M. B cell-helper neutrophils stimulate the diversification and production of immunoglobulin in the marginal zone of the spleen. Nat Immunol. (2011) 13:170–80. doi: 10.1038/ni.2194, PMID: 22197976 PMC3262910

[139] Kowalska W Bojarska-Junak A. Monocytic MDSC as a source of immunosuppressive cytokines in chronic lymphocytic leukemia (CLL) microenvironment. Folia Histochem Cytobiol. (2020) 58:25–36. doi: 10.5603/FHC.a2020.0006, PMID: 32227331

[140] Galletti G Caligaris-Cappio F Bertilaccio MT. B cells and macrophages pursue a common path toward the development and progression of chronic lymphocytic leukemia. Leukemia. (2016) 30:2293–301. doi: 10.1038/leu.2016.261, PMID: 27677742

[141] Sun L Guo RF Newstead MW Standiford TJ Macariola DR Shanley TP. Effect of IL-10 on neutrophil recruitment and survival after Pseudomonas aeruginosa challenge. Am J Respir Cell Mol Biol. (2009) 41:76–84. doi: 10.1165/rcmb.2008-0202OC, PMID: 19097982 PMC2701962

[142] Horn KJ Fulte S Yang M Lorenz BP Clark SE. Neutrophil responsiveness to IL-10 impairs clearance of Streptococcus pneumoniae from the lungs. J Leukoc Biol. (2024) 115:4–15. doi: 10.1093/jleuko/qiad070, PMID: 37381945 PMC10768920

[143] Wigerblad G Kaplan MJ. Neutrophil extracellular traps in systemic autoimmune and autoinflammatory diseases. Nat Rev Immunol. (2023) 23:274–88. doi: 10.1038/s41577-022-00787-0, PMID: 36257987 PMC9579530

[144] Vossenaar ER Zendman AJ van Venrooij WJ Pruijn GJ. PAD, a growing family of citrullinating enzymes: genes, features and involvement in disease. Bioessays. (2003) 25:1106–18. doi: 10.1002/bies.10357, PMID: 14579251

[145] Zhu D Lu Y Wang Y Wang Y. PAD4 and its inhibitors in cancer progression and prognosis. Pharmaceutics. (2022) 14:2414. doi: 10.3390/pharmaceutics14112414, PMID: 36365233 PMC9699117

[146] Li M Lin C Deng H Strnad J Bernabei L Vogl DT. A novel peptidylarginine deiminase 4 (PAD4) inhibitor BMS-P5 blocks formation of neutrophil extracellular traps and delays progression of multiple myeloma. Mol Cancer Ther. (2020) 19:1530–8. doi: 10.1158/1535-7163.MCT-19-1020, PMID: 32371579 PMC7335350

[147] Deng H Lin C Garcia-Gerique L Fu S Cruz Z Bonner EE. A novel selective inhibitor JBI-589 targets PAD4-mediated neutrophil migration to suppress tumor progression. Cancer Res. (2022) 82:3561–72. doi: 10.1158/0008-5472.CAN-21-4045, PMID: 36069973 PMC9532374

[148] Wang Y Wang M Zhang H Wang Y Du Y Guo Z. Sivelestat improves clinical outcomes and decreases ventilator-associated lung injury in children with acute respiratory distress syndrome: a retrospective cohort study. Transl Pediatr. (2022) 11:1671–81. doi: 10.21037/tp-22-441, PMID: 36345446 PMC9636449

[149] Cruz MA Bohinc D Andraska EA Alvikas J Raghunathan S Masters NA. Nanomedicine platform for targeting activated neutrophils and neutrophil-platelet complexes using an alpha(1)-antitrypsin-derived peptide motif. Nat Nanotechnol. (2022) 17:1004–14. doi: 10.1038/s41565-022-01161-w, PMID: 35851383 PMC9909445

[150] Bazak R Houri M El Achy S Kamel S Refaat T. Cancer active targeting by nanoparticles: a comprehensive review of literature. J Cancer Res Clin Oncol. (2015) 141:769–84. doi: 10.1007/s00432-014-1767-3, PMID: 25005786 PMC4710367

[151] Kirtane AR Kalscheuer SM Panyam J. Exploiting nanotechnology to overcome tumor drug resistance: Challenges and opportunities. Adv Drug Delivery Rev. (2013) 65:1731–47. doi: 10.1016/j.addr.2013.09.001, PMID: 24036273 PMC3849460

[152] Zhang L Gu FX Chan JM Wang AZ Langer RS Farokhzad OC. Nanoparticles in medicine: therapeutic applications and developments. Clin Pharmacol Ther. (2008) 83:761–9. doi: 10.1038/sj.clpt.6100400, PMID: 17957183

[153] Wilson DJ DuBois RN. Role of prostaglandin E2 in the progression of gastrointestinal cancer. Cancer Prev Res (Phila). (2022) 15:355–63. doi: 10.1158/1940-6207.CAPR-22-0038, PMID: 35288737 PMC9359060

[154] Amano H Eshima K Ito Y Nakamura M Kitasato H Ogawa F. The microsomal prostaglandin E synthase-1/prostaglandin E2 axis induces recovery from ischaemia via recruitment of regulatory T cells. Cardiovasc Res. (2023) 119:1218–33. doi: 10.1093/cvr/cvac137, PMID: 35986688 PMC10411941

[155] Domingo-Gonzalez R Martinez-Colon GJ Smith AJ Smith CK Ballinger MN Xia M. Inhibition of neutrophil extracellular trap formation after stem cell transplant by prostaglandin E2. Am J Respir Crit Care Med. (2016) 193:186–97. doi: 10.1164/rccm.201501-0161OC, PMID: 26417909 PMC4731709

[156] Wang D Dubois RN. Prostaglandins and cancer. Gut. (2006) 55:115–22. doi: 10.1136/gut.2004.047100, PMID: 16118353 PMC1856377

[157] Sollberger G Choidas A Burn GL Habenberger P Di Lucrezia R Kordes S. Gasdermin D plays a vital role in the generation of neutrophil extracellular traps. Sci Immunol. (2018) 3:eaar6689. doi: 10.1126/sciimmunol.aar6689, PMID: 30143555

[158] Adrover JM Carrau L Dassler-Plenker J Bram Y Chandar V Houghton S. Disulfiram inhibits neutrophil extracellular trap formation and protects rodents from acute lung injury and SARS-CoV-2 infection. JCI Insight. (2022) 7:e157342. doi: 10.1172/jci.insight.157342, PMID: 35133984 PMC8983145

[159] Ohms M Moller S Laskay T. An attempt to polarize human neutrophils toward N1 and N2 phenotypes *in vitro* . Front Immunol. (2020) 11:532. doi: 10.3389/fimmu.2020.00532, PMID: 32411122 PMC7198726

